# Promoting a more empowering motivational climate in physical education: a mixed-methods study on the impact of a theory-based professional development programme

**DOI:** 10.3389/fpsyg.2025.1564671

**Published:** 2025-06-24

**Authors:** Daniel Milton, Paul R. Appleton, Anna Bryant, Joan L. Duda

**Affiliations:** ^1^Cardiff School of Sport & Health Sciences, Cardiff Metropolitan University, Cardiff, United Kingdom; ^2^Institute of Sport, Manchester Metropolitan University, Manchester, United Kingdom; ^3^Cardiff School of Education & Social Policy, Cardiff Metropolitan University, Cardiff, United Kingdom; ^4^School of Sport, Exercise & Rehabilitation Sciences, University of Birmingham, Birmingham, United Kingdom

**Keywords:** *Empowering PE*, teacher training, achievement goal theory, self-determination theory, longitudinal design, pupil engagement, community of practice

## Abstract

This study examined the effects of the delivery of a school-tailored *Empowering PE*™ workshop and subsequent Professional Development Programme (PDP) using Community of Practice principles within one secondary school Physical Education (PE) department. Employing a mixed-methods longitudinal design, the research assessed PE teachers' understanding of motivation and motivational strategies, Senior Leadership Team perceptions of the PDP's impact, and pupils' motivation and engagement in PE. The intervention pulled from Achievement Goal Theory and Self-Determination Theory, adopting Duda's theoretically integrated conceptualization of empowering and disempowering motivational climates. Qualitative data from interviews, focus groups, and reflections revealed significant perceived benefits for teachers' understanding and implementation of motivational strategies, as well as perceived improvements in pupil engagement and motivation. Quantitative data (147 Year 9 pupils aged 13–14 years, M = 13.6, SD = 0.4; 81 boys, 64 girls) revealed no significant differences over time in perceptions of the motivational climate and their motivation to engage in PE. Overall, this study contributes to the growing body of evidence supporting the benefits of creating empowering environments in physical education. The findings however highlight the complexity of implementing and assessing the effects of motivational climate interventions in PE settings and underscore the importance of sustained, theory-informed professional development for teachers. Areas for future research on interventions (and testing their effectiveness) to optimize the motivation climate in PE, pupil motivation and teacher professional development are provided.

## Introduction

The motivational climate created by teachers in physical education (PE) settings plays a crucial role in shaping pupils' experiences, engagement, and overall motivation. Over the past few decades, research has consistently demonstrated the significant impact of teacher-created motivational climates on the quality of pupils' experiences in PE (Curran and Standage, 2017; Vasconcellos et al., 2020). It is apparent that teachers matter when it comes to the motivation of their pupils and whether their engagement is positive and adaptive or maladaptive and non-optimal (Havik and Westergård, 2020; Taylor et al., 2008). Consequently, supporting teachers to optimize the psychosocial environment in their classes, i.e., the motivational climate, to ensure pupils' motivation is of high quality and sustained could ensure all pupils in PE have increased positive experiences and play a critical role in delivering health benefits and enhancing physical activity (Mastagli et al., 2021). Teachers who promote the behaviors and strategies underlying an optimal environment for learning and engagement, where pupils are allowed to collaborate in their own development, are more likely to achieve positive learning outcomes and enhance quality forms of motivation (Noltemeyer et al., 2019; Núñez and León, 2015).

Despite this wealth of knowledge, there remains a pressing need to bridge the gap between theory and practice (O'Leary et al., 2015), particularly in developing effective interventions that can enhance teachers' ability to create optimal motivational climates. This study aims to address this gap by examining the effects of a novel, theoretically-grounded professional development program (PDP) on PE teachers' understanding and implementation of motivational strategies, as well as its impact on pupils' experiences and motivation in PE classes. By doing so, we seek to advance the field's understanding of how to effectively translate motivational climate theory into practice within PE settings.

The approach adopted to the conceptualization of the motivational climate in this study integrates two prominent theories of motivation: achievement goal theory (AGT; Nicholls, 1989) and self-determination theory (SDT; Ryan and Deci, 2017). While these theories have often been studied separately in PE contexts, recent work has highlighted the potential benefits of their integration in PE, performing arts and coaching settings (Hancox et al., 2017; Smith et al., 2015; Vasconcellos et al., 2020). In the present work, we adopt Duda's (2013) theoretically-integrated conceptualization of empowering and disempowering motivational climates as the conceptual foundation for the intervention.

SDT and AGT provide distinct but complementary perspectives on motivation and optimizing participation in educational settings (Deci and Ryan, 2000). SDT explains why people engage in activities and how different types of motivation lead to varying qualities of engagement (Deci and Ryan, 2000; Reeve, 2012). SDT posits that motivation exists on a continuum from amotivation to controlled motivation (extrinsic rewards/pressures) to autonomous motivation (intrinsic drive). Autonomous motivation, characterized by volition and personal endorsement, is associated with deeper engagement and persistence (Ryan and Deci, 2020). Recent work highlights how empowering climates satisfy basic psychological needs (autonomy, competence, relatedness), fostering autonomous motivation (Diloy-Peña et al., 2024) while Granero-Gallegos et al. (2024) has further clarified and operationalized this continuum in educational settings, demonstrating how empowering climates foster more autonomous forms of motivation and better teaching intentions. Specifically within in PE settings autonomous motivation is also associated with more positive engagement and other associated outcomes (Standage et al., 2005; Villavicencio and Bernardo, 2013).

SDT also posits that teachers' behaviors can either support or frustrate pupils' basic psychological needs for autonomy, competence, and relatedness which hold implications for the degree to which pupils' motivation is self-determined (Ryan and Deci, 2020). When these basic psychological needs are satisfied, individuals are more likely to exhibit personal growth, well-being and be autonomously motivated (Vasconcellos et al., 2020). Teachers who encourage pupil autonomy (e.g., offer meaningful choices and welcome pupils' input), provide clear explanations and informative feedback to enhance perceptions of competence, give encouragement and social support to promote feelings of relatedness, satisfy the basic psychological needs and subsequently, promote pupils' autonomous motivation (Aelterman et al., 2019). Conversely, controlling behaviors (such as using intimidation to force desired processes and outcomes; employing extrinsic rewards in a contingent manner) that thwart the psychological needs have been found to lead to more controlled forms of motivation or amotivation (Bartholomew et al., 2010), poorer engagement, and compromised wellbeing (González et al., 2017).

AGT focuses on how individuals define and judge competence in achievement contexts (Nicholls, 1989). It distinguishes between task-involving climates, which emphasize personal improvement and effort, cooperation, and learning from mistakes, from ego-involving climates. In an ego-involving motivational climate, the focus is on outperforming others, responses to undesirable performance tends to be punitive, and there is a rivalrous hierarchy (based on differences in ability) promoted between group/team members (Ames, 1992; Newton et al., 2000). Task-involving climates promote adaptive motivational outcomes, while ego-involving climates can lead to maladaptive responses, especially for less skilled individuals and/or when heightened challenges are presented to the capable (Duda and Balaguer, 2007; Duda et al., 2014).

Pulling from both AGT and SDT, Duda's (2013; Duda et al., 2024) framework defines more empowering motivational climates as those marked by autonomy supportive, task-involving, and social supportive leader behaviors. Conversely, disempowering climates are assumed to be characterized by controlling and ego-involving behaviors. This theoretically integrated approach provides a more comprehensive understanding of how teachers can create motivationally supportive environments in PE, fostering pupils' autonomous motivation, engagement, and positive experiences (Mageau and Vallerand, 2003; Aelterman et al., 2019). They can do this by employing more strategies and interacting for pupils in a more empowering way, thus reducing disempowering behaviors. While research has examined the effects of interventions based on either AGT (e.g., Braithwaite et al., 2011; Morgan and Carpenter, 2002) or SDT (e.g., Cheon et al., 2016; Llanos-Muñoz et al., 2022; Sánchez-Oliva et al., 2017) principles in PE, studies have begun to pull from Duda's integrated conceptualization of the motivational climate in educational settings (e.g., Girard et al., 2021). Overall, previous research findings provide preliminary support for the value of combining the key constructs and tenets of SDT and AGT when investigating and looking to modify the empowering and disempowering features of the teacher-created motivational climate (Weeldenburg et al., 2021).

Research has demonstrated that teacher created empowering environments hold implications for pupils' emotional states and impacts the quality of learning in educational contexts (Hancox et al., 2017). Mastagli et al. (2021) investigated the relationship between an empowering motivational climate and pupils' concentration and distraction in PE. Their findings suggest that PE teachers can enhance pupils' motivation and concentration while reducing distraction by supporting the development of pupils' competence, providing clear structures and expectations, planning for specific skill development, and focusing on effort and individual progress. These elements reflect characteristics of an empowering motivational climate, highlighting that such a climate not only improves pupils' engagement but also suggests that such a motivational environment can promote cognitive functioning during PE classes. In terms of intervention efforts, Girard et al. (2021) examined the effects of a training course they developed which aimed to create an empowering motivational climate in PE. Their quasi-experimental study demonstrated that PE teachers who underwent specific training were better equipped to create such a climate. The researchers concluded that fostering an empowering climate is not only desirable but also achievable through targeted professional development.

A limitation of psychosocial interventions within PE settings has often been their failure to address the complexities of implementing sustained change in educational settings (Armour et al., 2017). More recent research on the effectiveness of professional development programs (PDPs) has suggested that embedding theoretical concepts alongside a more sustained and collaborative approach is vital in promoting the success and quality of interventions (Braga et al., 2017). Therefore, reshaping the traditional “one-off” workshop into PDPs that are ongoing, research-informed, and collaborative is vital (Yoon and Armour, 2017). Tannehill et al. (2021) suggest that PDPs for teachers should be centered on teacher needs, that teachers should be active collaborators who gain pedagogical skills and content knowledge and are supported with time and care. One approach to developing such PDPs is to implement collaborative principles from the community of practice (CoP) literature (Lander et al., 2020). Recent interventions using SDT-based teacher training have shown promise in enhancing pupil engagement (Wang et al., 2024), while CoP models have demonstrated sustainability in PE contexts (Ocete et al., 2025).

### Theoretical integration for motivational climate interventions

This study integrates AGT's task/ego climate distinction with SDT's need-support framework through Duda's (2013) empowering/disempowering model. CoP principles operationalize this integration by fostering collaborative, sustained teacher development. The development and utilization of CoPs have emerged as an effective approach for facilitating professional learning among (PE) teachers. Research has shown that CoPs provide PE teachers with a platform for collaborative learning, knowledge sharing, and enhancing their teaching skills and effectiveness (MacPhail et al., 2014; Parker et al., 2022). By engaging in CoPs, PE teachers can develop pedagogical innovations, share practical teaching ideas, and receive appropriate support from peers and facilitators (Goodyear and Casey, 2015; Armour and Yelling, 2004; Parker et al., 2010). These benefits of CoPs offer a strong rationale for their incorporation into professional development programs for PE teachers. Previous research has suggested that this has already been successful in CPD training centered on promoting more adaptive motivational climates, where teachers have become more confident and empowered to create autonomy-supportive climates (Braga et al., 2017).

The present study builds on the existing professional development literature, taking a collaborative PDP approach using the principles of CoP to support school staff to create more empowering motivational climates. In the realm of motivational climate research, recent studies have begun to explore the potential of technology-enhanced interventions (e.g., Lander et al., 2020) and the role of school-wide approaches to creating positive motivational climates (e.g., Claver et al., 2020). In addition, Simon et al. (2025) found anxiety/pleasure dynamics modulate concentration in empowering climates, while Milton et al. (2025a,b) highlight motivation's mediating role in engagement. These developments underscore the need for interventions that can adapt to changing educational landscapes while maintaining a strong theoretical foundation.

The present research is grounded Duda's (2013) theoretically integrated empowering/disempowering model of the motivational climate and incorporates a novel PDP approach to facilitate the creation of a more empowering motivational climate in secondary school PE classes. The intervention employs a sustained PDP approach, incorporating principles of CoP to support long-term implementation of more empowering strategies and reflection on current and forthcoming practice (Goodyear and Casey, 2015). A mixed-methods design was chosen to capture both teacher experiences (qualitative) and pupil outcomes (quantitative), allowing triangulation of intervention effects across stakeholder levels (Creswell and Plano Clark, 2007). Qualitative approaches have proven critical in unpacking teachers' motivational strategies (White et al., 2021), informing this study's focus on lived experiences of PE staff. We employed a mixed-methods evaluation of the intervention's impact on both teachers and pupils over time. The study considers not only changes in pupil-focused outcomes but also shifts in teachers' understanding and application of motivational strategies, providing insight into the mechanisms of change. More specifically, the purpose of this study was to examine the effects of a school tailored *Empowering PE*™ workshop and subsequent PDP using the principles of CoP within a secondary school PE department with the following objectives: (1) via qualitative methods, PE teachers' understanding of motivation and optimal and dysfunctional motivational strategies, and reported motivational strategies employed within the school day, (2) via qualitative methods, Senior Leadership Team members' (SLT) perceptions of the PE teachers understanding, engagement and impact of the PDP, and (3) via the administration of established questionnaires, pupils' perceptions of the motivational climate (empowering and disempowering), motivation and indicators of their engagement within PE.

## Methods

This study employed a longitudinal mixed-methods approach to examine the effects of a school-tailored *Empowering PE*™ workshop and subsequent professional development programme (PDP) using the principles of CoP within one secondary school PE department. The research design was chosen to address the study's multifaceted aims, which required both in-depth qualitative exploration and quantitative measurement of change over time.

### Research design

**Figure 1:**
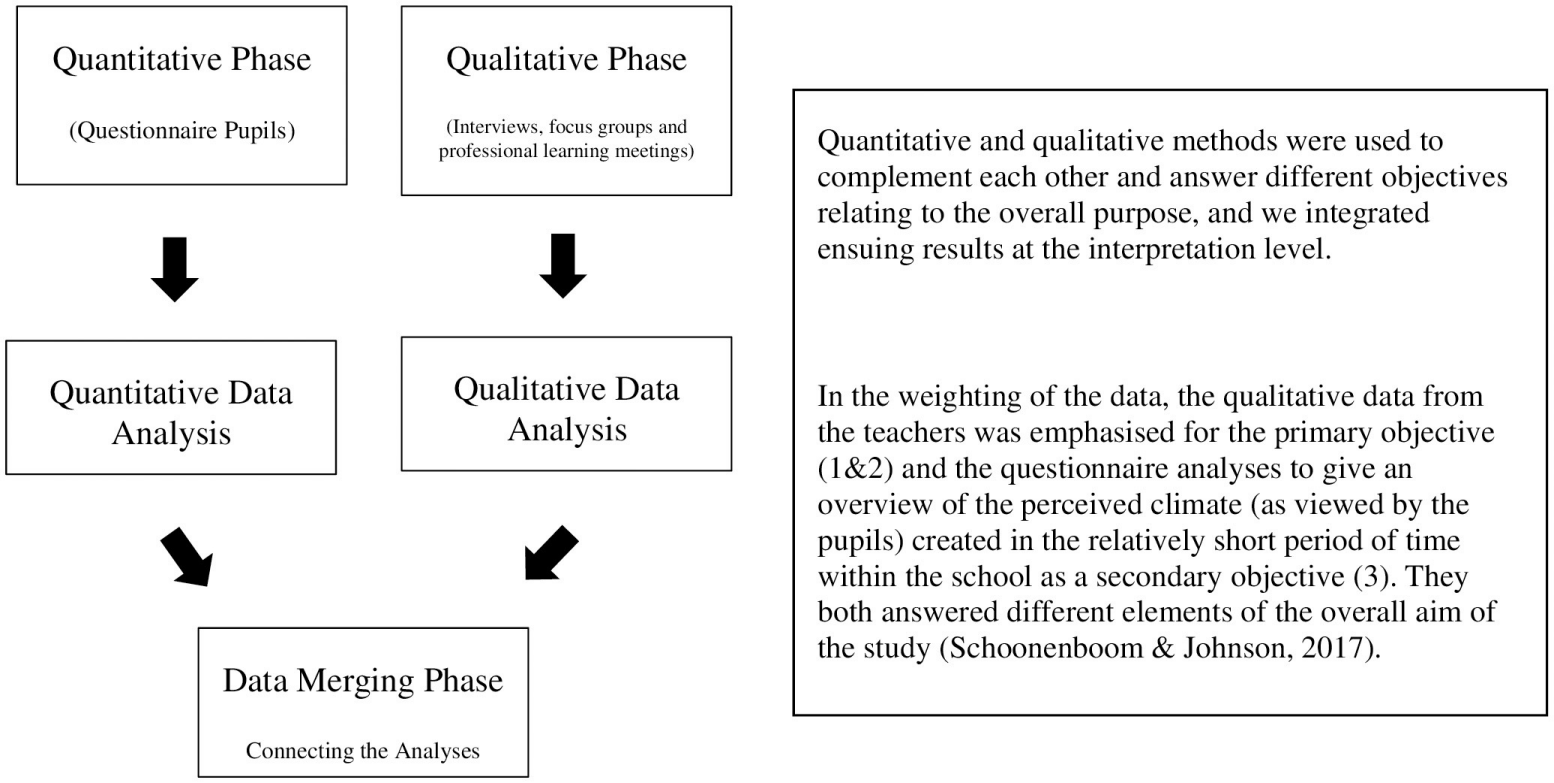
Concurrent/parallel research design.

A pragmatic philosophical stance underpinned the mixed-methods design, reflecting the study's focus on addressing real-world educational challenges (Van der Roest et al., 2015). This approach aligns with Mertens' (2007) recommendation for researchers to be explicit about their philosophical perspective in mixed-methods research. A convergent parallel mixed-methods design was employed, with qualitative (interviews/focus groups) and quantitative (questionnaires) data collected concurrently to triangulate findings (Schoonenboom and Johnson, 2017). The mixed-methods design enabled us to triangulate findings and prioritize qualitative insights where quantitative data may be less sensitive to change or distributional nuances. The longitudinal design, spanning eight months (July 2019–February 2020), was essential to capture the processes of change within the school context, as advocated by Hargreaves and Goodson (2006). The rationale for employing a mixed-methods approach was threefold: Firstly, we wanted to include methodological diversity. The combination of quantitative and qualitative approaches allowed for a more comprehensive inquiry into the complex phenomena of motivational climate change and teacher professional development (Creswell and Plano Clark, 2007; Tashakkori and Teddlie, 2009); Secondly, we sought to realize complementarity and triangulation. The integration of quantitative and qualitative data enabled direct comparison and contrast of results, validating and expanding both datasets (Creswell, 2010). This approach enhanced the depth and breadth of understanding regarding the study's key elements within the PE context (König, 2016). Thirdly, the adoption of a mixed methods approach allowed us to address multiple research questions. The mixed-methods design was deemed conducive to the exploration of different aspects of the study's overall aim, capturing perspectives from teachers, senior leadership, and pupils through a concurrent design. Figure 1 below illustrates the approach taken to weighting and mixing within this study.

### Data collection and integration

The study utilized multiple data sources, both qualitative and quantitative, to address its purpose and objectives: Qualitative data were gathered through multiple sources: pre- and post-intervention interviews with the Headteacher and Senior Leadership Team (SLT) in July 2019 and February 2020; pre- and post-intervention focus groups with the PE Department during the same time periods; ongoing teacher reflections throughout the Professional Development Programme (PDP); researcher's voice memos; professional learning conversations; discussions via WhatsApp; and one-year follow-up interviews with the PE Department in April 2021. Quantitative data were collected through pupil questionnaires administered pre-intervention in September 2019 and post-intervention in February 2020.

**Figure 2:**
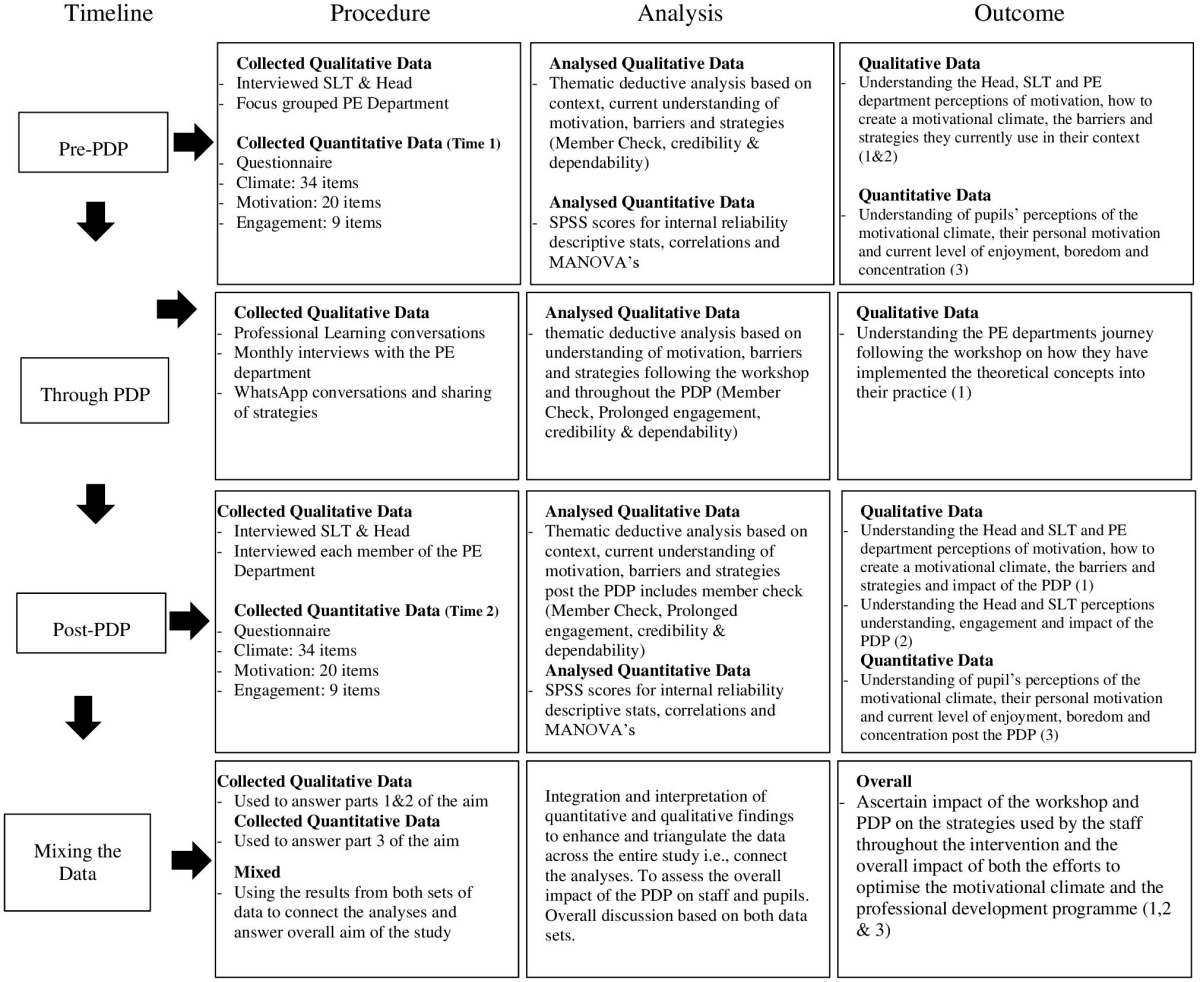
Visual model of the timeline, data collection and data analysis process.

This allowed for an examination of the intervention's impact from multiple perspectives over an extended period. The integration of these data sources followed Creswell's (2009) recommendations for mixed-methods research, considering timing, weighting, mixing, and theorizing/transforming. Data collection was concurrent, with equal weight given to qualitative and quantitative strands. The mixing of data occurred at the interpretation stage, following Schoonenboom and Johnson's (2017) guidelines for parallel mixed analysis. For a visual model of the timeline please see Figure 2 and all data collection tools available on request.

### Participants and setting

Ethics committees from the authors' two universities approved the project and the school and teachers were invited to participate in the study. The study was conducted at one comprehensive secondary school in Wales, UK. Participants included: two Headteachers (both male), two senior leaders (both female), PE Department (two male and two female PE teachers with between 5 and 20 years teaching experience) and 147 Year 9 pupils (13 and 14 years old, 81 boys and 64 girls). This diverse sample allowed for a comprehensive exploration of the intervention's impact across different levels of the school hierarchy and among the target pupil population.

### Instruments and procedures

#### Qualitative methods

Semi-structured interviews and focus groups were conducted pre- and post-intervention. These methods allowed for detailed information gathering while maintaining interviewer control over the data received (Armour and MacDonald, 2012). The interview and focus group protocols were designed to ensure credibility and consistency across different time points and participant groups (Creswell, 2010). Interview questions explored participants' understanding of motivation, motivational climate, and strategies used to motivate pupils. The Headteachers and SLT interviews provided insights into their perceptions of changes in teaching quality and pupil experience (duration of interviews varied from 45 to 90 mins).

Throughout the PDP, ongoing conversations and discussions via WhatsApp supported the development of strategies and understanding of theoretical content. WhatsApp was used as a platform for informal support and the sharing of conversations and strategies as requested by the teachers within the PDP. Boundaries were discussed around the use of WhatsApp and the appropriate timings and volume of messages were considered and modified throughout. All interviews, focus groups, and professional learning conversations were audio-recorded and transcribed verbatim. Online conversations were documented in writing. Participant identities were protected using pseudonyms.

#### Quantitative methods

A multi-section questionnaire was administered to pupils pre- and post-intervention. The questionnaire assessed pupils' perceptions of the teacher-created motivational climate, their motivation to do PE, and reported engagement-related outcomes capturing their experiences in PE lessons. The measures all used Likert scales from 1 to 5, and took ~20 min to complete, included:

1. Empowering and Disempowering Motivational Climate Questionnaire-Physical Education (EDMCQ-PE; Milton et al., 2018), Dimensions: Autonomy Support, Social Support, Task involving, Ego Involving and Controlling.
2. Perceived Locus of Causality Questionnaire (PLOCQ; Goudas et al., 1994), Dimensions: Autonomous (intrinsic and identified) and Controlled (introjected and external).
3. Satisfaction Interest Scale (SIS; Duda and Nicholls, 1992), Dimensions: enjoyment, boredom and concentration.

These validated scales were chosen for their relevance to the study's theoretical framework and their previous use, reliability and validation in PE contexts. While means and standard deviations were reported for Likert-scale data, we recognize that such summary statistics may not fully capture the distributional properties of ordinal responses (e.g., potential bi-modality). This limitation is inherent in much survey research and should be considered when interpreting the findings (see also Norman, 2010). Despite this limitation, the use of means and MANOVA is common in educational psychology research and allows for comparability with previous studies (Field, 2018; Pallant, 2020). The quantitative trends were used to complement in-depth qualitative analysis to provide a more nuanced interpretation of the results.

### Intervention

The intervention consisted of a multi-component PDP for PE teachers, beginning with a theory-informed and evidence-based workshop developed by Duda (2013; namely *Empowering P*E™). The workshop, adapted for the PE context (see Duda, 2013; Duda et al., 2024), were delivered by the first author, who had both theoretical expertise and practical experience in PE teaching (see Table 1). The workshop content was divided into three 2.5-h sessions to minimize disruption to school staffing and thus, hopefully maximize participation.

**Table 1:** Content of workshops within phase 2.

**Workshop 1**	**Workshop 2**	**Workshop 3**
• Introduction to the training • Philosophy and setting of initial individual and departmental goals • Understanding the quality and quantity of motivation • Collaborative discussion using applied examples	• Introduce the ABC's Autonomy Belonging and Competence • Teachers' generation of empowering strategies Co-operative contribution Learning emphasized Intrinsic focus Mastery oriented Authority with autonomy Taking other's perspectives Evaluation	• Recap the theoretical concepts within the workshop • Collaborative discussion on theory to practice • Introduce the concept of a PDP using principles of CoP outlining the potential benefits and creating the boundaries and placing the author as the ‘boundary spanner'

Following the initial workshop, the PDP continued using principles of CoP (For a detailed review on the development of the principles please see Milton et al., 2025a) to facilitate ongoing training and embedding of strategies. This approach included regular professional learning meetings, online discussions, and collaborative problem-solving sessions. Several recommendations to build effective CPDs and CoPs have been implemented in various educational settings including PE (Armour et al., 2017; Edwards et al., 2019; De Carvalho-Filho et al., 2020). With the strategies and recommendations from previous studies in mind (see Table 2), we employed those which were applicable to the context in question in this study (Milton et al., 2025a).

**Table 2:** Strategies of effective CPD and CoPs and application to this study (adapted from Armour et al., 2017; Edwards et al., 2019; Trust and Horrocks, 2019; De Carvalho-Filho et al., 2020).

	**Strategies used**	**Application to this study**
A	In depth needs assessment	*Qualitative needs assessment of the Head Teacher, SLT and PE Department*
B	Gather a core group to launch the process	*Staff members of the PE Department with whole school support*
C	Start with a specific task or project embedded with theory and applied to practice:	*understanding and implementation of motivation and empowering motivational strategies*
D	Co-develop the purpose of the community with the members, giving them voice and choice with how they learn	*create individual and departmental goals shared and created by the participants*.
E	Create sustainable support structures, opportunities to collaborate and increase social learning	*establish 3–4-week touch points to review, reflect and shape the next cycle*
F	Use technology to support and connect	*Use the participants to come up with a way of online sharing that would engage and help sustain the group i.e., WhatsApp*
G	Make it worthwhile for members and the institution	*Evidence the learning and development throughout, build and share strategies including success and failure. Work it into their schedule – make it work for them*

### Data analysis

The study employed a mixed-methods analysis design (Tashakkori and Teddlie, 2009), analyzing data at multiple levels to incorporate individual components (König, 2016).

#### Qualitative analysis

Thematic saturation guided qualitative data collection, achieved when no new codes emerged across three consecutive interviews. Participant numbers aligned with recommendations for in-depth case studies (Braun and Clarke, 2019). Deductive thematic analysis (Patton, 2015) was used to analyse the qualitative data, an approach chosen due to the study's grounding in existing theory (i.e., Duda's, 2013 conceptualization), which provided the lens through which the data was interpreted (Braun and Clarke, 2019). The analysis process, adapted from the six-step approach outlined by Braun and Clarke (2006), involved several key stages. First, interviews and focus groups were transcribed and anonymised. Initial coding was then conducted based on whether the data were collected: (a) Pre-PDP or (b) During and Post-PDP. Within each higher-order theme, subthemes were developed. The research team engaged in iterative analysis and discussion to refine the themes. Finally, illustrative quotes were selected to support each theme. To enhance trustworthiness, the lead researcher engaged in reflexive practice, acknowledging their personal and social standpoint within the research. Such reflexive practice is considered to be crucial in qualitative research (Finlay and Gough, 2008). The deductive themes and codes were discussed with the wider research team to ensure credibility and reduce potential bias, aligning with recommendations for enhancing rigor in qualitative analysis (Nowell et al., 2017). Triangulation used multiple data sources (interviews, focus groups, WhatsApp logs). Member checking involved participant feedback on themes. Procedures followed Tracy's (2010) guide for qualitative research emphasizing the importance of rigor, sincerity, and meaningfulness in qualitative inquiry.

#### Quantitative analysis

The analysis process involved several steps to ensure data quality and extract meaningful insights. First, data screening for normality and outliers was performed following guidelines by Tabachnick and Fidell (2007). This involved examining histograms, boxplots, and z-scores to identify potential outliers. The reliability of the scales was assessed using Cronbach's alpha coefficients. Following established guidelines, an alpha value exceeding 0.80 was considered to indicate high reliability (Clark and Watson, 1991). For scales with more than 10 items, an alpha of 0.70 was deemed acceptable, while for scales with fewer than 10 items, a lower threshold of 0.60 was considered acceptable (Hair et al., 2010). The Primary outcomes were: empowering/disempowering climate (EDMCQ-PE), autonomous/controlled motivation (PLOCQ) while the Covariates: age, gender. Analyses controlled for baseline scores.

Descriptive statistics, including means and standard deviations, were computed for all variables at both time points to provide an overview of the data. To examine relationships between variables, Spearman rank-order correlations were conducted, as this non-parametric test is suitable for ordinal data or when normality assumptions are violated (Field, 2018). Finally, repeated measures MANOVAs were performed to assess changes in variables between time points, allowing for the evaluation of the intervention's impact over time (Pallant, 2020). All analyses were conducted using IBM SPSS Statistics 26 (George and Mallery, 2019).

#### Integration of qualitative and quantitative analyses

Following the separate analyses of qualitative and quantitative data, the results were integrated to provide a more multi-faceted understanding of the intervention's impact. This integration process involved several key steps. Themes emerging from the qualitative data were compared with trends observed in the quantitative results, allowing for a more nuanced interpretation of the findings. Areas of convergence and divergence between the qualitative and quantitative findings were identified, highlighting both consistencies and potential discrepancies in the data. The rich contextual information provided by the qualitative data was used to explain and contextualize the quantitative results, offering deeper insights into the observed statistical patterns. Finally, a cohesive narrative was developed that synthesized both data types, addressing the study's research questions and providing a holistic view of the intervention's effects.

### Trustworthiness and validity of the study

Several strategies were employed to ensure the rigor and trustworthiness of the study, drawing on Lincoln and Guba's (1985) criteria for trustworthiness in qualitative research. These include (1) Prolonged engagement: The researcher spent eight months within the school setting, ensuring a deeper understanding of the context and enhancing credibility, (2) Member checking: Conversations (within interviews, focus groups, and discussions) were shared with participants to verify the accuracy of findings (Creswell and Plano Clark, 2007), (3) Dependability: The researcher maintained detailed records of thoughts, methods, and decisions through research voice memos and discussions with the research team. (4) Triangulation: Multiple data sources (interviews, focus groups, questionnaires, and ongoing discussions) were used to corroborate findings, (5) Peer debriefing: Regular discussions with the research team helped refine codes, themes, and interpretations. For quantitative data, validity was considered using previously validated scales and reliability via examination of internal consistency of each scale (Cronbach's alpha).

## Results

### Qualitative results

Addressing aim 1, we ascertained PE teachers' understanding of motivation and optimal and dysfunctional motivational strategies, and reported motivational strategies employed within the school day, and 2. Senior Leadership Team's (SLT) perceptions of the PE teachers understanding, engagement and impact of the PDP. Analyses were conducted based around the timelines of the data collection (see Table 3): 1. Pre the PDP, which was comprised of two themes (i.e., Importance of motivation and lack of understanding, and Current strategies on developing motivation), and 2. During and post the PDP, which were comprised of three themes (i.e., Strategies tried through the PDP and improvement in teaching methods, Impact of the PDP on Pupils, Impact of the PDP on the Staff and School).

**Table 3:** Initial coding and themes.

**Core codes**	**Subthemes**	**Higher order themes (timelines)**
Progress	Importance of motivation and lack of understandin	Pre the PDP
What is success		
Wellbeing		
Lack of understanding of motivation		
Lack of training on motivation		
Assessment Focused Role to inspire, encourage and enthuse		
Control/Fear	Current strategies of developing motivation		
Rewards		
Pupil Voice		
Rankings		
Disengagement		
Acknowledging people		
Modeling behaviors		
Relationships		
Mixed Ability		
Use of Language	Strategies tried through the PDP and improvement in teaching methods	During and Post the PDP
Wellbeing		
Task focused view of competence		
Variety		
Use of pupil voice		
Use of Questions		
Groupings		
Planning		
Pupils taking more responsibility	Impact of the PDP on Pupils		
Engaging teaching styles		
Belonging through language and relationships		
Understanding different contexts		
Research informed: The ABC's	Impact of the PDP on the Staff and School		
Thought provoking		
Trial and Error		
Stretch and challenge		
Longevity, ongoing engagement		

### Pre-PDP theme 1: importance of motivation and lack of understanding

A critical finding was the widespread lack of specific training and understanding of motivation and motivational climate among staff at all levels, from the Headteacher to the (PE) teachers. This gap in knowledge was particularly striking given that staff universally recognized their role as educators to “challenge”, “enthuse”, “inspire”, and foster enjoyment in school for both pupils and colleagues. The Headteacher, Stan, exemplified this disconnect, stating “I think their [teachers] role is to help encourage and enthuse…I think what they need to do as a classroom teacher is to enthuse pupils for that thing that made you love your subject.” But when queried on what specific training teachers receive in this area, a typical response was “Nothing! It's only what I would have learned when I was a pupil doing an undergraduate degree in sport in college and then using that knowledge to apply to situations in an educational setting”. This sentiment was echoed by other staff members, including Anthea from the SLT, who admitted: Do you know—very little...I would say I've had very little to none, in terms of specifically on motivation, or how you kind of motivate pupils. PE teacher Sandra further highlighted this gap:

We've done lots of stuff on teaching and learning, we've done nothing on motivational climate. The climate of your classroom—how do you stimulate an individual through your planning…how do you plan a lesson for an individual to come in and be motivated—that climate of stimulation? I wouldn't say we've done any of that, in my ten years here (Sandra, PE Teacher)

This finding underscores a significant disparity between the recognized importance of motivation in education and the actual targeted preparation and education teachers receive to effectively create more adaptive (empowering) motivating learning environments and understand variability in pupils' motivation.

The pre-PDP interviews and focus groups also revealed that staff were eager to enhance their understanding of motivation and the motivational climate, particularly in relation to their teaching practices. This enthusiasm was especially evident in their desire to improve their use of language and strategies for engaging disengaged pupils. A key insight emerged from Karen's perspective, highlighting a fundamental misunderstanding about the relationship between behavior, learning environment, and motivation: “you need to have the positive behavior and the positive kind of learning environment first and foremost before you can actually work on that motivation aspect”. This statement underscores a critical gap in understanding: the interconnectedness of motivational climate, behavior, and learning environment. It reveals the need for teachers to recognize that creating an empowering motivational climate can itself positively influence behavior and the learning environment, rather than being a separate or consequential step.

The analysis further uncovered that teachers' conceptualization of motivational climate lacked specificity. Terms like “positive behavior” and “positive learning environment” were used vaguely, without clear definitions or sound and systematic practical applications. This finding emphasizes the importance of providing teachers with (and facilitating them in developing) concrete, actionable strategies for creating empowering motivational climates. Adrian's reflection on his language used with pupils exemplifies the unintentional use of disempowering strategies:

“I think it's quite easy to say the wrong thing as a teacher, because you get wrapped up in the moment... I've said to someone 'If you don't get a C your mother's not going to be happy and you're not going to get into college'... It's awful because you get wrapped up in the moment of them not working and you're like 'how can I just get you to work?' So, I think having the correct language to use at the right time; knowing what strategies to use when, what can I put in place to get them back on task”.

### Pre-PDP theme 2: current strategies on developing motivation

Analyses revealed the widespread use of extrinsic, controlling rewards and strategies across the school, which were often ineffective or even counterproductive in motivating pupils. One particular example of a misguided motivational strategy was the use of public ranking systems:

One strategy was ranking kids, and they thought that would motivate them. They published past papers, or they did tests and then they ranked the whole year group, and it was up on a board outside the classroom. We had huge issues from that… I remember the head of science coming to me, saying parents were phoning in, saying “How dare you! My kid's on the bottom” and the head of science was like “Well, you're on the bottom because you're not working hard enough”. Those sorts of things. That did not work at all. And you can see why, can't you? But at the time the head of science was like “Well, you want to be top of the pile”. (Karen, SLT)

This quote illustrates how well-intentioned but poorly conceived motivational strategies can backfire, causing distress among pupils and parents while failing to achieve the desired motivational outcomes. Karen's example, using the head of science's response to complaints, further demonstrates a lack of understanding about effective motivational approaches.

Another critical finding is the teachers' struggle to create an empowering motivational climate, particularly when introducing new or challenging activities. This is exemplified by Adrian's experience with teaching rugby:

“We did rugby last year, and in one class half the girls really wanted to do it and the other half didn't, and the other half that didn't really ruined the climate of the class; because it just crept into the people not doing it and it affected how the ones that wanted to do it, could play”. (Adrian, PE Teacher)

This quote emphasizes the complex interplay between pupil preferences, activity choice, and the overall motivational climate of the class. It underscores the need for teachers to develop strategies that can engage all pupils and create an empowering learning environment, even when introducing activities that may initially be met with resistance.

### During and post PDP theme 1: strategies tried through the PDP and improvement in teaching methods

The data revealed that the Professional Development Programme (PDP) resulted in significant changes in the PE staffs' language, perceptions, and ability to apply motivational strategies in practice. Staff found the workshops impactful and thought-provoking, providing practical ideas and strategies. Karen (SLT) noted, 'Workshops have been really positive…ideas and strategies…that's been a really good thing…staff enjoy ideas they can go and put into practice.' Sandra echoed this sentiment, describing the material as 'excellent' and 'very research based.' Teachers demonstrated a greater grasp of motivational theory and its application in lessons. Alan (HOD), for example, reported a significant shift toward emphasizing belonging and autonomy:

“From my perspective as a teacher, I've had a real emphasis on belonging and autonomy—being focused on task-oriented behaviors; where the pupils have come up with their own ideas about tasks...It's had huge, huge gains in empowerment, in terms of pupils just taking ownership of a lesson, and even their body language, their relationships with their peers, have been very effective.”

Teachers began adopting empowering strategies that promoted pupil autonomy and engagement. Adrian, a PE teacher, described successfully adapting suggested activities:

“I nicked one of the activities you suggested, which was to ask them to come up with a game, or an activity...I used the activity to allow them a broad interpretation of it and allowed pupils to just enjoy the game, I was able actually to intervene and say, 'How do we challenge that?”

The PDP led to improvements in teachers' communication styles, benefiting pupil relationships. Adrian noted a significant reduction in negative interactions with pupils:

“I only thought about it in my personal wellbeing; if I hadn't screamed at a kid I'd feel a lot better, a lot happier...I've thought about it in my progress leader role, just how I've been speaking to kids, the interaction, because as soon as I start negative, negative, negative, that kid just shuts off. I've been trying to open up conversations, to build that relationship.”

The sustained nature of the PDP allowed for continuous refinement and adaptation of strategies. Adrian's experience with different year groups illustrates this process:

“Year 7′s autonomy, (I feel) they wanted to be told...Whereas there was massive success with the Year 9s. They loved it, and they were far more engaged, the pupil teacher was watching it as well and he couldn't believe how engaged they were, quite complex drills they came up with.”

A critical outcome was the observed improvement in teaching methods and motivational climate as the PDP progressed. The Headteacher noted tangible changes in teachers' language and approach, observing more positive, motivational language and increased pupil autonomy in lessons: “very motivational, positive language…there was constant reassurance of how things could be done and how well it was going...almost scaffolding the learning and allowing pupils to go off and be independent” (Tony, Interim Head). The researcher's reflections highlighted the value of the PDP for collaborative learning and exchange amongst the staff members themselves:

“I think what struck me today was the value of the PDP… the interaction. It's the reaction of the department who are, as a basis of practices that have been posted or examples shared, the conversations. It's driving, shaping some of the things that they're doing, it's driving communications between the staff members; it's become the forefront of what they're doing.”

### During and post PDP theme 2: impact of the PDP on the pupils

PE Teachers reported significant improvements in pupil engagement and performance, particularly among typically disengaged pupils. Alan (HOD) remarked on the “remarkable” impact of emphasizing autonomy on pupils who were not stereotypically sporty or interested in football: “What I saw by certain pupils was remarkable. I know that's a strong word. Pupils who are not stereotypically sporty or enjoy football. It was crazy by giving them or emphasizing (autonomy) had a significant impact on the motivation and quality of work”. Adrian provided a specific example of successfully engaging a previously unmotivated Year 9 class in football through positive language and increased pupil autonomy:

“I had a rotation with Year 9 girls for football in January…they were very de-motivated and didn't want to play. But after using positive language, giving praise, and allowing them autonomy to create their own drills, they became really engaged and motivated. They've absolutely loved it”.

Taken in their totality, the qualitative findings summarized above support the effectiveness of the theoretically informed, school-tailored *Empowering PE*™ workshop and subsequent PDP using Community of Practice principles. The intervention contributed to an increased understanding of motivation and optimal motivational climates, development and the implementation empowering strategies among teachers, along with perceived positive impacts on pupil motivation and behavior. Additionally, the intervention was deemed to have a positive impact on the teaching staff and school. Overall, it facilitated a shift toward more effective and sustainable professional development practices in physical education teaching.

### During and post PDP theme 3: impact of the PDP on the teachers

The qualitative results supported the impact of the Professional Development Programme (PDP) on teachers' understanding and implementation of motivational strategies in physical education (PE). A key finding was the perceived value and engagement with the PDP across all levels of staff. The Headteacher and Senior Leadership Team (SLT) noted high levels of engagement and longevity in the staff's participation. Tony, the Interim Head, highlighted ‘that staff enjoyed the workshops and were able to immediately apply new ideas and strategies in their teaching'. Andrea (SLT) reinforced

“they've had with you on a personal level and the meetings they've had with you. They seem to have been fully engaged with the research project, and I think that has been because they've seen an impact on what they've been doing as well. They've been very positive in terms of what they've been trying to trial and put in place. They've been having positive kind of feedback from pupils with that. I think that helped to keep them going”.

The PDP was seen as having a positive impact on individual teachers' professional development and motivation to teach. Alan, a PE teacher, expressed that the programme had “regenerated his understanding of what excellent teaching and learning looks like…reigniting my passion for teaching…refreshing for the department”. Similarly, Sandra noted that the PDP prompted her to critically reflect

‘I've been teaching for 10 years and it's just really made me have a look at my teaching, if anything you get stuck in a groove and you do the same things day in, day out…just thinking why I'm doing things and is that the right way to do it, just because I've always done it that way?'

The longevity and varied interaction methods of the PDP contributed to its sustainability. Teachers appreciated the ability to “dip in and out” of the programme, with regular posts and reminders keeping them focused and encouraging innovative thinking

Allowed us to dip in and out…It was very thought-provoking, but it was tending toward what each of us needed (Alan, HOD)'

“(the PDP) kept it fresh, there were people posting daily, weekly, or when there was a reminder from you. So, as I said, it kept me focused, kept me thinking a little bit outside the box”. (Adrian, PE Teacher)

This ongoing engagement contrasts sharply with traditional one-off workshops. Even a year after the intervention, despite disruptions from the Covid-19 pandemic, the impact persisted. Alan (HOD) confirmed continued impact on practice and noted that the department had evolved in terms of sharing practice and embracing the concept of a community of practice: 'To answer the impact one, yes. There is still an impact in practice…I think we have certainly evolved as a department in sharing practice and looked at the idea of a community of practice being more than just CPD'

### Quantitative results: pupils' perceptions of the motivational climate, motivation and targeted outcomes reflecting engagement

Data screening procedures were adopted to detect outliers and normality in the sample in line with guidelines from Tabachnick and Fidell (2007). The internal consistency (see Table 4) estimates (α) for all the measures ranged from 0.73 to 0.91, indicating acceptable reliability. The quantitative results (see Table 4) revealed several noteworthy findings regarding the “status quo” regarding pupils' perceptions of the motivational climate, their motivation, and engagement in PE. Prior to the PDP (see Table 4), pupils already perceived the teacher-created motivational climate to be more empowering than disempowering. The mean scores for empowering climate (3.96 at Time 1) were moderately high, while disempowering climate scores (2.66 at Time 1) were moderately low. In addition, pupils reported moderately high levels of autonomous motivation (M = 3.88) and enjoyment (M = 3.96), with concentration also relatively high (M = 3.85). Notably, controlled motivation was moderately high (M = 2.93), suggesting that external pressures or rewards still played a significant role in pupils' motivation. In contrast, boredom levels were relatively low (M = 2.29), indicating that pupils generally found their PE classes engaging or at least not boring. These baseline scores suggest that while pupils experienced more positive motivational states overall, there was still room for improvement, particularly in reducing disempowering strategies and controlled motivation and further enhancing empowering motivational behaviors autonomous motivation in physical education classes.

**Table 4:** Internal consistency, means and SD for time 1 and time 2 samples.

**Variable**	**Time 1 (** ***N*** **: 147)**			**Time 2 (** ***N*** **: 144)**		
	**M**	**SD**	α	**M**	**SD**	α
1 Empowering	3.96	0.56	0.90	3.93	0.58	0.92
2 Disempowering	2.66	0.51	0.78	2.58	0.52	0.81
3 Autonomous Motivation	3.88	0.97	0.94	3.95	0.95	0.94
4 Controlled Motivation	2.93	0.84	0.80	2.84	0.74	0.73
5 Enjoyment	3.96	0.99	0.89	4.07	0.92	0.91
6 Concentration	3.85	0.96	0.86	3.90	0.95	0.89
7 Boredom	2.29	1.08	0.82	2.31	1.00	0.79

Bivariate correlations revealed that pupils' perceptions of empowering climates were positively related to autonomous motivation, enjoyment and concentration and negatively correlated to controlled motivation and boredom (see Table 5). Perceived disempowering climates were positively related to controlled motivation and boredom and negatively related to autonomous motivation, enjoyment and boredom. Also consistent with Duda's (2013) framework, perceptions of empowering and disempowering climates were significantly and negatively correlated.

**Table 5:** Bivariate correlations for time 1 and time 2.

**Variable (time 1 *N*:147)**	**2**	**3**	**4**	**5**	**6**	**7**
1 Empowering	−0.27^**^	0.54^**^	−0.10	0.59^**^	0.56^**^	−0.36^**^
2 Disempowering		−0.11	0.33^**^	−0.18^*^	−0.19^*^	0.32^**^
3 Autonomous motivation			−0.06	0.86^**^	0.80^**^	−0.54^**^
4 Controlled motivation				−0.12	−0.08	0.39^**^
5 Enjoyment					0.80^**^	−0.62^**^
6 Concentration						−0.55^**^
7 Boredom						
**Variable (Time 2** ***N*****:147)**	**2**	**3**	**4**	**5**	**6**	**7**
1 Empowering	−0.29^**^	0.71^**^	−0.16	0.72^**^	0.67^**^	−0.46^**^
2 Disempowering		−0.14	0.13	−0.14	−0.11	0.11
3 Autonomous motivation			−0.16	0.85^**^	0.80^**^	−0.56^**^
4 Controlled motivation				−0.19^*^	−0.11	0.46^**^
5 Enjoyment					0.81^**^	−0.56^**^
6 Concentration						−0.54^**^
7 Boredom						

* *p* < 0.05.

** *p* < 0.01.

### Changes in pupils' scores on targeted variables

Repeated measures MANOVAs were conducted to examine whether differences existed between participants' scores on the targeted scales from time point one (pre-intervention) to time point two (post intervention). Findings revealed no significant differences over timepoints 1 and 2, and the contrast results all moved through 0 on the confidence intervals (see Table 6). Results were as follows: empowering and disempowering [Wilks' lambda = 0.96, F_(1.729)_ = 2.00, *p* = 0.12 η_p_2 = 0.02]; autonomous and controlled motivation [Wilks' lambda = 0.99, F_(0.82)_ = 2.00, *p* = 0.41, η_p_2 = 0.01] and enjoyment, concentration and boredom [Wilks' lambda = 0.99, F_(0.67)_ = 3.00, *p* = 0.57 $\eta_{\text{p}}^{2}$ = 0.01].

**Table 6:** Contrast results for MANOVAS.

**95% confidence interval for difference**	**Empowering**	**Disempowering**	**Autonomous Motivation**	**Controlled Motivation**	**Enjoyment**	**Concentration**	**Boredom**
Lower bound	−0.52	−0.05	−0.31	−0.09	−0.33	−0.26	−0.25
Upper bound	0.21	0.19	0.13	0.29	−0.11	0.18	0.24

### Mixing the data analyses

The quantitative and qualitative analyses answered different elements of the study's overall purpose. Mixing methods and subsequent analyses can contribute new insights into this study's specific context and overall findings. Table 7 outlines the key learnings from each set of results so it is possible to explicitly relate the quantitative and qualitative information (McCrudden et al., 2019). These findings underscore the complexity of implementing and measuring the impact of motivational climate interventions in PE settings. While the qualitative data suggests positive changes in teacher understanding and practices and motivation and engagement of the pupils, the quantitative results indicated that these changes may not have immediately translated into significant improvements in pupil perceptions of the climate, motivation, and engagement.

**Table 7:** Mixing the data analyses.

**Overall purpose: examine the effects of a school tailored** ***Empowering PE***™ **workshop and subsequent professional development programme (PDP) within one secondary school PE department in Wales**			
**Qualitative**	**Key learnings following the PDP**	**Quantitative**	**Key learnings following the PDP**
(1) Teachers' understanding of motivation and motivational climate	1. Pre-PDP: Teachers lacked specific training and understanding of motivation and motivational climate concepts. 2. Post-PDP: Teachers developed and implemented strategies to enhance motivation and create more empowering climates. 3. Impact: Positive effects on teaching practices, staff wellbeing, and perceived pupil motivation. 4. Sustainability: The PDP's sustained approach was perceived as more effective than traditional CPD methods.	(2) Pupils' perception of the motivational climate, their motivation and quality of engagement.	1. Minimal improvements in pupils' perceptions of motivational climate and motivation following the PDP. 2. Slight decreases in disempowering climate perceptions and controlled motivation. 3. Small increases in autonomous motivation, enjoyment, and concentration. 4. No statistically significant differences between Time 1 and Time 2 measurements.
**Critical observations**			
1. Disconnect between qualitative and quantitative results: teachers reported significant improvements, but quantitative data showed only minimal, non-significant changes. 2. Potential limitations in the sensitivity of quantitative measures or the duration of the intervention. 3. The importance of mixed methods approaches in capturing nuanced changes in educational interventions. 4. Need for longer-term follow-up to assess sustained impact on pupil outcomes.			

## Discussion

This study examined the effects of a school-tailored *Empowering PE*™ workshop and subsequent PDP using CoP principles within a secondary school PE department. The research aimed to assess 1. PE teachers' understanding of motivation and motivational strategies, 2. Senior Leadership Team (SLT) perceptions of the PDP's impact, and 3. perceptions of the motivational climate (empowering and disempowering), quality of pupils' motivation and indicators of engagement within PE. The findings extend previous research suggesting CPD education workshops could enhance PE teachers' understanding of why pupil motivation and the motivational climate are important and how empowering and disempowering strategies impact their pupils (Milton et al., 2018). As such, the results of this study were expected to contribute to the growing body of evidence supporting the benefits of creating empowering environments in PE (Girard et al., 2021).

### Empowering climates and impact on pupils' motivation

The qualitative findings revealed that the intervention produced perceived benefits for pupils' motivation and engagement, supporting previous studies that have demonstrated the positive impact of task-focused, social belonging, and autonomy-focused strategies on pupils' motivation (Behzadnia et al., 2018; Leisterer and Jekauc, 2019; Vasconcellos et al., 2020). Teachers reported improvements in teaching and learning, wellbeing, and increased autonomous motivation in both pupils and staff. These results align with Sevil-Serrano et al.'s (2020) work on developing features of the motivational climate within a school (or in this case, department) to influence teachers' strategies. The findings of this study align with and extend previous research on empowering climate strategies in PE. For example, our results are consonant with Behzadnia et al. (2018) findings on the positive impact of autonomy-supportive teaching on pupil motivation. However, our study goes further by demonstrating how a sustained PDP can help teachers develop and implement these strategies effectively over time. Additionally, the findings on the perceived benefits for pupil wellbeing and peer relationships contribute to the growing body of evidence on the broader impacts of empowering motivational climates (e.g., Vasconcellos et al., 2020; Leisterer and Jekauc, 2019).

The quantitative results showed no significant improvements in pupils' perceptions of the motivational climate and motivation following the PDP across the two time points. The observed discrepancy between qualitative and quantitative findings highlights the complexity of implementing and measuring motivational climate interventions in PE settings. It also underscores the importance of using mixed methods approaches to capture nuanced changes in educational interventions (McCrudden et al., 2019).

The lack of significant quantitative changes (in the pupils' perspectives) could be due to several factors. First, the relatively short intervention period may not have allowed sufficient time for changes in teacher behavior to translate into measurable shifts in pupil perceptions. Second, the sensitivity of the quantitative measures used may not have been adequate to capture subtle changes in the motivational climate and ensuing motivational processes. Future studies could consider using more sensitive or tailored instruments to detect smaller changes over time, such as Multidimensional Motivational Climate Observation System (MMCOS; Smith et al., 2015; Tzoumaki et al., 2025). We assessed before and after the delivery of the *Empowering PE*™ workshop and CoP-based PDP but it is important to note that the assessments were secured at the beginning and end of the academic year. So there could be other factors in play (exams, fatigue, anticipation of holidays) impacting the pupils' responses. These limitations suggest the need for longer-term studies with larger sample sizes and more frequent data collection points to better capture the dynamic impact of such interventions (Stenling et al., 2017) and these effects may not be linear (Tzoumaki et al., 2025). Moreover, in the present study, we did not have a control group to help couch the present findings in regard to change from T1 to T2. Perhaps, for example, pupils whose teachers did not receive such an intervention exhibit significant changes in the targeted variables... but those changes were not positive. In the present study, at Time 2, the motivation and engagement of the pupils in this study were not diminished. Nor did the motivational climate created by the PE teachers become more maladaptive.

### Sustained theoretically informed PDPs

A key finding of this study is the effectiveness of theory-informed workshops combined with a tailored PDP in developing teachers' theoretical knowledge and practical application. This supports Braga et al. (2017) emphasis on the importance of establishing research-informed CPD in PE. The sustained nature of the intervention, using CoP principles, allowed for ongoing reflection, adaptation, and embedding of concepts in teachers' practice. This approach addresses Armour et al. (2017) call for more effective CPD methods that go beyond traditional one-off workshops.

The study's use of Duda's (2013) integrated conceptualization of AGT and SDT within a sustained PDP represents a relatively novel contribution to the field. By combining these theoretical perspectives and applying them to a real-world educational setting, the research offers a more comprehensive approach to understanding and influencing motivational climates in PE. The findings of this study have important implications for educational policy and practice beyond PE. The success of the theory-informed, sustained PDP approach, as garnered from the qualitative suggests that similar models could be effective in other subject areas and educational contexts.

### Characteristics of effective CPD

The findings support recent literature on effective PDPs, emphasizing the importance of focusing on pedagogic principles (Morgan et al., 2018), fostering learning communities (Tannehill et al., 2021), and recognizing the complexity of planning CPDs (Yoon and Armour, 2017). The study's results provide Armour, Makopoulou and Chambers (2015) recommendations to recognize the “dazzling complexity” of PE teacher development and bridge theory to practice in innovative ways. The use of technology, particularly the WhatsApp platform, to support the PDP aligns with the growing importance of digital tools in education (Lander et al., 2020). This aspect of the intervention demonstrates the potential for technology to enhance the sustainability and accessibility of professional development initiatives, a finding that has become even more relevant since the COVID-19 pandemic.

### Implications for theory, research, and practice

This study advances the understanding of motivational climate interventions in PE and has important implications for theory, research, and practice globally. By using Duda's (2013) integrated conceptualization of AGT and SDT, the study provides a more nuanced and detailed understanding of motivational climates in PE settings. This integration of theories offers a more comprehensive framework for future research, supporting the growing trend of combining AGT and SDT perspectives in sport and PE motivation studies (Duda and Appleton, 2016). The combination of workshops with an ongoing PDP using CoP principles offers a model for more effective and sustainable professional development in PE but also in regard to how to optimse motivational climate interventions in general. The sustained, tailored approach adopted addresses limitations of traditional one-off workshops, which often lack long-term impact (Armour et al., 2017).

The study's mixed-methods design, incorporating both qualitative and quantitative methods, provided a more comprehensive evaluation of the intervention's impact. This approach addresses calls for more innovative research designs in PE motivation studies (De Meester et al., 2020) and allows for a deeper understanding of the complex relationships between motivational climates and pupil outcomes (Van den Berghe et al., 2014). By including multiple perspectives from teachers, Senior Leadership Team members, and pupils, the study offers a holistic understanding of the intervention's effects. This multi-level approach aligns with recent recommendations for examining the impact of motivational climates in PE (Curran and Standage, 2017).

The identified lack of prior training (and misunderstanding) on motivation and motivational climates among teachers highlights a need for greater emphasis on theory and theoretically-grounded motivational strategies in initial teacher education programs internationally. This finding suggests that professional learning opportunities focused on creating empowering motivational climates should be incorporated into teacher preparation and induction programs (Braithwaite et al., 2011). From a policy perspective, the alignment between empowering motivational climates and broader educational goals, such as fostering ambitious and capable learners, suggests that policymakers should consider embedding these principles into curriculum reforms and teacher development initiatives. This could have far-reaching implications for enhancing pupil motivation and engagement in PE and beyond (Reeve, 2012).

Finally, the mixed results between qualitative and quantitative data underscore the need for more sophisticated research methodologies, particularly longitudinal designs, to better capture the nuanced effects of interventions over time. Future studies should consider employing extended timeframes and multiple data collection points to more accurately assess the impact of motivational climate interventions on pupil outcomes (Stenling et al., 2017) and allow for the possibility that any observed effects are not linear (Tzoumaki et al., 2025).

### Limitations and future directions

Transparency is critical in documenting the study's limitations, methods, and analytic procedures, this was achieved using best practice recommendations for mixed-methods research (Creswell and Plano Clark, 2007). Several limitations must be acknowledged in this study, as an overview the quantitative data had a relatively small sample which limited statistical power, while the qualitative data had the potential for self-report bias. Both quantitative and qualitative data have a single-school focus which potentially affects generalizability. In more detail, the small sample size and limited quantitative data collection points restrict the generalisability of findings and the ability to detect significant changes (whether linear, quadractic) over time. Additionally, the relatively short duration of the intervention may have constrained observable effects. Focusing on a single school in one region of Wales further limits the applicability of the results; however, this focus allowed for an in-depth exploration of the context. Future research should aim to replicate this study across multiple schools and diverse settings, seeking larger samples and more frequent data collection over a longer period of time to better capture the intervention's impact. Longitudinal studies spanning multiple years could provide a more comprehensive understanding of the long-term benefits of such interventions.

Future research could also explore the use of more objective measures of teacher behavior (Smith et al., 2015) and pupil outcomes (Reeve, 2012). Employing classroom observations or performance assessments could complement self-report data and offer a more robust evaluation of teaching effectiveness. Investigating potential differential effects of the intervention on various pupil subgroups—such as by gender, ability level, or socioeconomic status—could yield valuable insights for tailoring motivational strategies to meet diverse pupil needs. Finally, it is important to consider that potential bias introduced by teachers' commitment to the project and their desire for success may have influenced their perceptions and responses. Future research should include more objective measures of teacher behavior and pupil outcomes to enhance the validity of findings.

## Conclusion

The current research makes a significant contribution to the literature on motivation, motivational climates, and longitudinal mixed methods studies in PE. By examining changes over a six-month period across multiple levels of analysis, this study provides evidence supporting the development of empowering strategies for teachers and their subsequent impact on pupils' motivation in PE. This study is the first to investigate an intervention designed to enhance teachers' understanding of motivational climate strategies using Duda's integrated conceptualization of AGT and SDT. The use of a theory-informed workshop followed by a sustained Professional Development Programme using Community of Practice principles represents a novel contribution to the literature on Continuing Professional Development in education. The mixed-methods longitudinal design employed in this study offers a more nuanced understanding of the intervention's impact, addressing the need for more sophisticated research designs in PE motivation studies. This approach allowed for the capture of both immediate and longer-term effects of the intervention on teachers' practices and pupils' experiences. While the study's findings are promising, future research should aim to replicate these findings in diverse educational contexts and with larger samples and more nuanced and repeated quantitative measures to enhance generalisability and detect potential changes in targeted variables. However schools could consider adopting CoP-based PDPs with 6-month timelines, integrating weekly peer reflections and quarterly workshops. Finally as a result policy initiatives could mandate motivation theory in teacher training curricula.

## Data Availability

The raw data supporting the conclusions of this article will be made available by the authors, without undue reservation.
